# High Temperature Fatigue of Aged Heavy Section Austenitic Stainless Steels

**DOI:** 10.3390/ma15010084

**Published:** 2021-12-23

**Authors:** Hugo Wärner, Guocai Chai, Johan Moverare, Mattias Calmunger

**Affiliations:** 1 Department of Management and Engineering, Linköping University, 58183 Linköping, Sweden; guocai.chai@sandvik.com (G.C.); johan.moverare@liu.se (J.M.); mattias.calmunger@liu.se (M.C.); 2 AB Sandvik Materials Technology R and D Center, 81181 Sandviken, Sweden

**Keywords:** high temperature austenitic stainless steels, out-of-phase thermomechanical fatigue, crack propagation analysis, electron backscatter diffraction (EBSD), energy-dispersive X-ray spectroscopy (EDS)

## Abstract

This work investigates two austenitic stainless steels, Sanicro 25 which is a candidate for high temperature heavy section components of future power plants and Esshete 1250 which is used as a reference material. The alloys were subjected to out-of-phase (OP) thermomechanical fatigue (TMF) testing under strain-control in the temperature range of 100 ${}^{\circ }$C to 650 ${}^{\circ }$C. Both unaged and aged (650 ${}^{\circ }$C, 3000 h) TMF specimens were tested to simulate service degradation resulting from long-term usage. The scanning electron microscopy methods electron backscatter diffraction (EBSD) and energy dispersive spectroscopy (EDS) were used to analyse and discuss active failure and deformation mechanisms. The Sanicro 25 results show that the aged specimens suffered increased plastic straining and shorter TMF-life compared to the unaged specimens. The difference in TMF-life of the two test conditions was attributed to an accelerated microstructural evolution that provided decreased the effectiveness for impeding dislocation motion. Ageing did not affect the OP-TMF life of the reference material, Esshete 1250. However, the structural stability and its resistance for cyclic deformation was greatly reduced due to coarsening and cracking of the strengthening niobium carbide precipitates. Sanicro 25 showed the higher structural stability during OP-TMF testing compare with the reference material.

## 1. Introduction

The global increase in energy consumption and the increase in emissions of greenhouse gases (e.g., CO_2_) causing global warming, make needs for both an increase in energy production and a reduction in greenhouse gas emissions [1]. One way to accomplish both needs is to increase the efficiency of biomass-fired power plants, which could be reached by increasing temperature and pressure in the boiler sections and consequently in other components of the system, such as headers and piping [2]. The heavy section materials used as headers and piping in the future high-efficient biomass-fired power plants are required to display improved properties, such as higher creep and fatigue strength and high-temperature steam oxidation resistance [3,4]. From previous studies [5,6], advanced austenitic stainless steels (A-ASS) materials, such as Sandvik Sanicro™ 25 (Sanicro 25), have shown to be a promising candidate. In addition, they are more cost-effective compared to nickel-based alloys that are another candidate for components in high-efficient biomass-fired power plants. Biomass-fired power plants are supposed to withstand many start and stop cycles to maintain efficiency in the global energy and electricity network [7,8]. This advocates for investigations of how cyclic operation conditions influence the high-temperature performance of heavy section A-ASS. Evaluation of the cyclic high-temperature properties can be performed by studying the thermomechanical fatigue (TMF) behaviour [9]. For a heavy section component, temperature gradients are problematic since it creates problems related to thermal expansion, where the expansion close to stress concentrators is often constrained by the cooler material in its surroundings. The thermal strain is then converted into mechanical strain which induces material damage. TMF testing in an out-of-phase (OP) condition shows a similar temperature and mechanical profile that simulates the real component condition for a hot-spot surrounded by colder material or the hot-side of a thicker wall with a temperature gradient [10,11]. OP-TMF corresponds to a material that undergoes compression at maximum temperature, $T_{max}$, and tension at minimum temperature, $T_{min}$. The reversed set-up, with maximum temperature during tension and minimum temperature in compression, is termed in-phase (IP) TMF. The potential and behaviour of A-ASS in power plant applications with TMF characteristics have, to some extent, already been evaluated [12,13,14,15,16,17,18]. From the studies of Nagesha et al. and Kumar et al. [12,13] (ΔT = 300–650 ${}^{\circ }$C), Petras et al. [14,15] and Polák et al. [16] (ΔT = 250–700 ${}^{\circ }$C), the IP-TMF condition was generally associated with grain boundary cracking originating from creep damage and oxidation due to the depletion of chromium caused by precipitation of chromium carbides. The OP condition was more susceptible to the surrounding environment, which yielded oxide surface cracking and transgranular propagation. Overall, the OP condition showed longer TMF life than the IP condition. Ageing applied before TMF cycling negatively affected the TMF lives of the austenitic stainless steels Sanicro 25, Sanicro 31HT (alloy 800H), and Esshete 1250 according to the IP-TMF (ΔT = 100–800 ${}^{\circ }$C, with 5 min dwell time at $T_{max}$) study by Wärner et al. [18]. The decrease in TMF lives was caused by embrittlement of the grain boundaries due to the microstructural evolution with extensive precipitation during ageing and creep related damage at the grain boundaries. Furthermore, in a study of relative low mechanical strain range OP-TMF testing (ΔT = 100–800 ${}^{\circ }$C) [17], it was found that aged austenitic alloys can suffer from inhomogeneous deformation over the test specimen gauge length (barrelling effect). Consequently, special care have to be taken when designing the specimen geometry to make it on the conservative side in reference to “Validated Code-of-Practice for Strain-Controlled Thermo-Mechanical Fatigue Testing” [19].

This work presents an investigation of the OP-TMF behaviour of the two austenitic stainless steels Sanicro 25 and Esshete 1250. Sanicro 25 is a potential candidate for future power plant heavy section components and the reference material Esshete 1250, is currently used for high-temperature power plant applications. The investigation includes evaluation of the material mechanical properties, combined with a microscopic analysis focusing on describing and linking the mechanical behaviour with the microstructural evolution.

## 2. Materials and Methods

**Figure 1:**
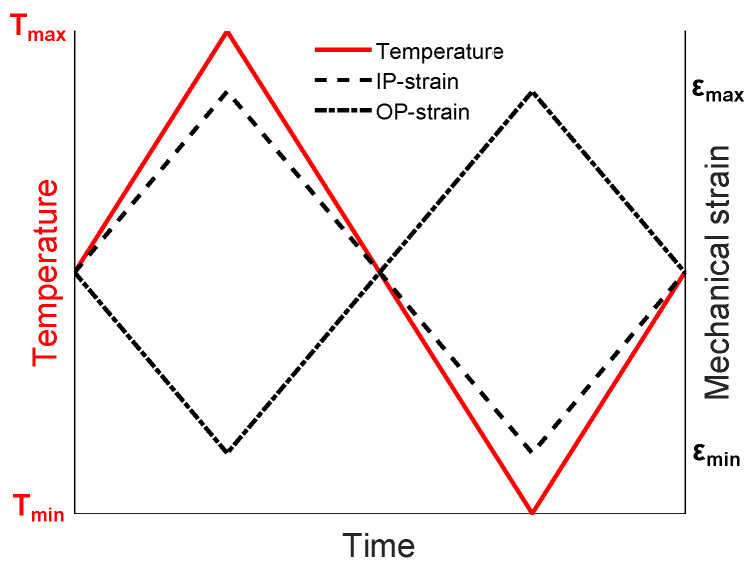
Schematics of the different TMF test cycles.

**Figure 2:**
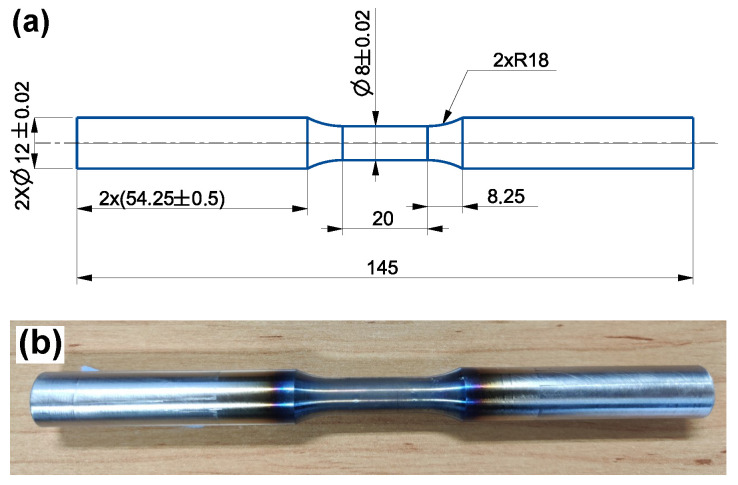
The test specimen, (**a**) schematics (units in millimetres), (**b**) after testing of an OP-TMF Ehsshete 1250 specimen.

The two austenitic stainless steels, Sanicro 25 (solution heat-treated at 1220 ${}^{\circ }$C for 10 min) and Esshete 1250 (solution heat-treated at 1100 ${}^{\circ }$C for 15 min), were studied and Sandvik Materials Technology AB provided and heat-treated the materials. In Table 1, the chemical compositions of the investigated materials in wt.% are given (provided by Sandvik Materials Technology AB). Sanicro 25 had an average initial grain diameter of 30 μm, but larger grains up to 192 μm were seen in the microstructure and Esshete 1250 had an average initial grain diameter of 32 μm, but larger grains up to 109 μm were seen in the microstructure. The used testing procedure was strain controlled TMF with the OP cycle and R_ε_ = −1 (see Figure 1 for the schematic TMF test cycles). The test machine was a servo-hydraulic TMF machine from Instron with induction heating and forced air-cooling. Before the TMF tests the machine was carefully aligned, to prevent buckling and other instability effects, according to the “Validated Code-of-Practice for Strain-Controlled Thermo-Mechanical Fatigue Testing” [19]. The temperature range, ΔT, was 100 ${}^{\circ }$C to 600 ${}^{\circ }$C, 650 ${}^{\circ }$C (main) and 700 ${}^{\circ }$C, the mechanical strain rate, $\dot{\varepsilon }$_mech_, was 0.01%/s and the heating and cooling rate was 5 ${}^{\circ }$C/s. From a half cylinder, exposed to hot extrusion, the test specimens were carefully machined according to Figure 2a,b and some specimens were aged at 600 ${}^{\circ }$C, 650 ${}^{\circ }$C (main), or 700 ${}^{\circ }$C (at $T_{max}$ of ΔT) for 3000 h before machining in order to simulate microstructural degradation from extended service time. The set-up of the tests were performed according to [19], with spot welded thermocouples aligned in the longitudinal direction, at a distance of 1mm between each other and the strain was measured with a high-temperature extensometer with a gauge length of 12.5 mm. The thermal strain was subtracted from the measured total strain, so that the mechanical strain ($\varepsilon_{mech}$) could be controlled as suggested by [19]. The number of cycles to failure (N_f_) was defined as the point where the stress range (Δσ) decreases 10% [19]. However, the test was not stopped until a load drop of 60%. Longitudinal cross sections of the tested specimens were ground and polished following the procedure used in [18]. The microstructural investigations were performed with a HITACHI SU-70 field emission gun (FEG)-scanning electron microscope (SEM) equipped with a solid-state backscattered electron (BSE) detector, using 10 and 20 kV acceleration voltage and working distances between 7.5 mm and 20 mm. The main used analysis techniques were electron backscatter diffraction (EBSD), in order to study the grain size, number and morphology, and energy-dispersive spectroscopy (EDS) to determine the composition of the present phases/precipitates. A Struers DuraScan G5, following ISO 6507, equipped with a Vickers diamond was used for the HV5 hardness measurements performed at room temperature of the TMF tested specimens.

## 3. Results

**Figure 3:**
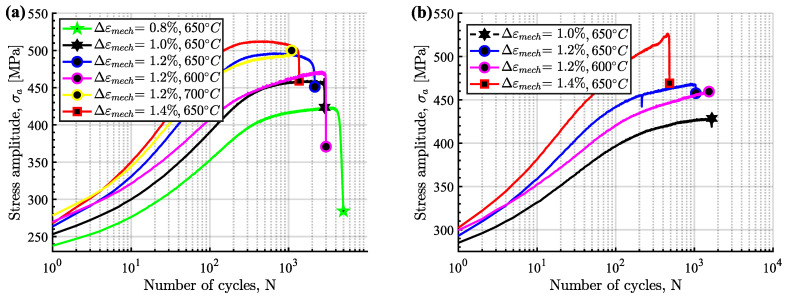
OP-TMF hardening curves of Sanicro 25, (**a**) unaged, (**b**) aged.

**Figure 4:**
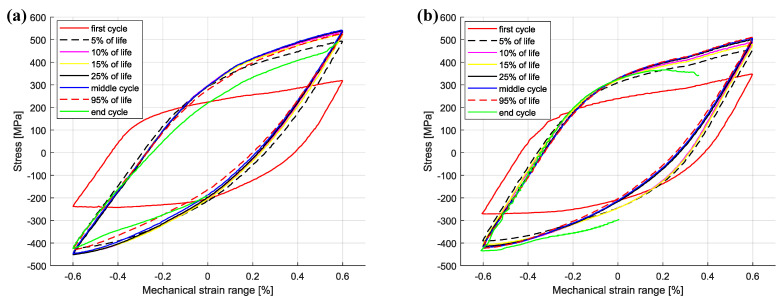
OP-TMF hysteresis curves of Sanicro 25, ΔT = 100–650 ${}^{\circ }$C, $\Delta \varepsilon_{mech}$ = 1.2%, (**a**) unaged, (**b**) aged.

**Figure 5:**
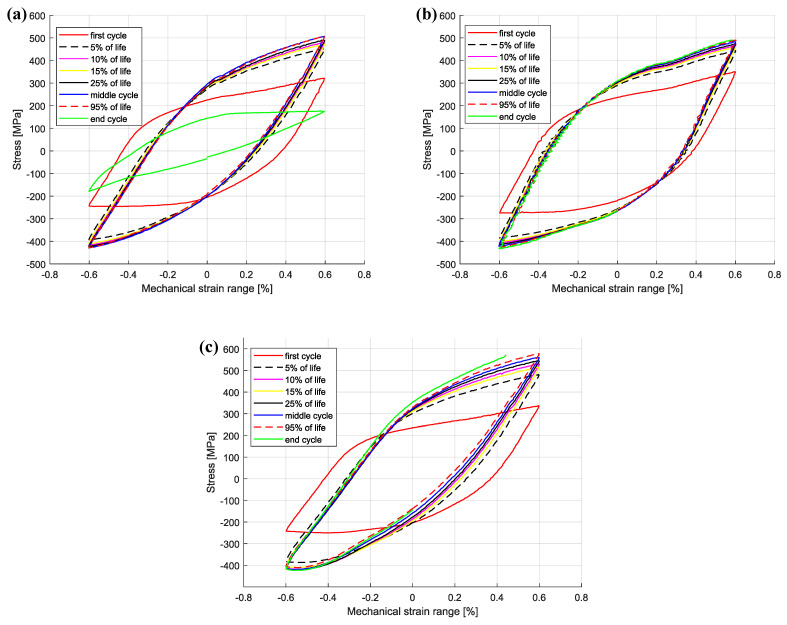
OP-TMF hysteresis curves of Sanicro 25, $\Delta \varepsilon_{mech}$ = 1.2%, (**a**) unaged, ΔT = 100–600 ${}^{\circ }$C, (**b**) aged, ΔT = 100–600 ${}^{\circ }$C, (**c**) unaged, ΔT = 100–700 ${}^{\circ }$C.

**Figure 6:**
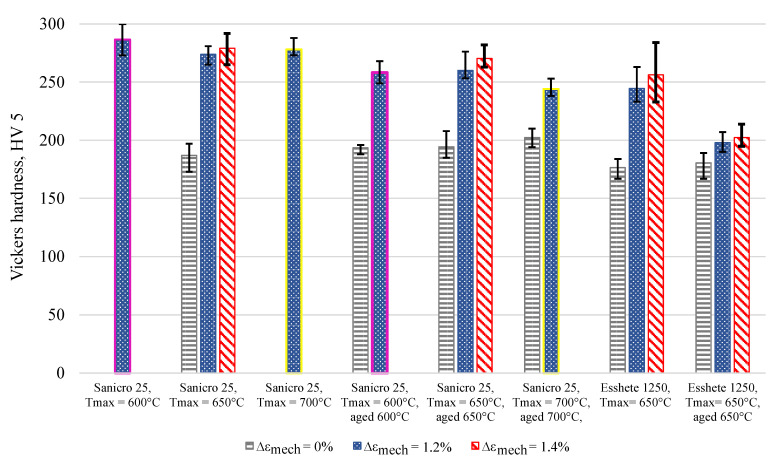
Vickers hardness of the investigated OP-TMF specimens (testing performed at room temperature after TMF cycling).

The cyclic hardening/softening curves of Sanicro 25 in unaged and aged condition are displayed in Figure 3 and they show that all the Sanicro 25 tests are associated with prominent initial hardening. However, the unaged specimens (Figure 3a) also showed stress saturation followed by softening until the load drop criteria was fulfilled and lower $\Delta \varepsilon_{mech}$ yielded both longer saturation and softening towards the end of the TMF life. In contrast, the aged Sanicro 25 specimens (Figure 3b) showed hardening until fracture occurred and overall fewer cycles to failure compared to the equivalent unaged specimens. In addition, the aged specimens experienced increased plastic strain range ($\Delta \varepsilon_{p}$), comparing Figure 4a and Figure 4b at the same percentage of life. In terms of fatigue life, altering the test temperature for the $\Delta \varepsilon_{mech}$ = 1.2% specimens approximately corresponded to changing the $\Delta \varepsilon_{mech}$ one step (1.0% for 600 ${}^{\circ }$C and 1.4% for 700 ${}^{\circ }$C). These reference $T_{max}$ tests (Figure 5) show similar difference of the plastic strain range as for the 650 ${}^{\circ }$C tests, with increased $\Delta \varepsilon_{p}$ for the aged specimen (Figure 5b) compared to the unaged specimen (Figure 5a). However, comparing Figure 5a and Figure 5c, the $T_{max}$ = 600 ${}^{\circ }$C and the $T_{max}$ = 700 ${}^{\circ }$C tests show similar stress and strain evolution. Considering the Vickers hardness result in Figure 6, these tests (Sanicro 25: $T_{max}$ = 600 ${}^{\circ }$C and $T_{max}$ = 700 ${}^{\circ }$C) show slightly lower hardness for the aged condition. This discrepancy is not evident for the $T_{max}$ = 650 ${}^{\circ }$C Sanicro 25 tests, but the initial state (undeformed specimens) all corresponds to lower hardness compared to the tested specimens.

**Figure 7:**
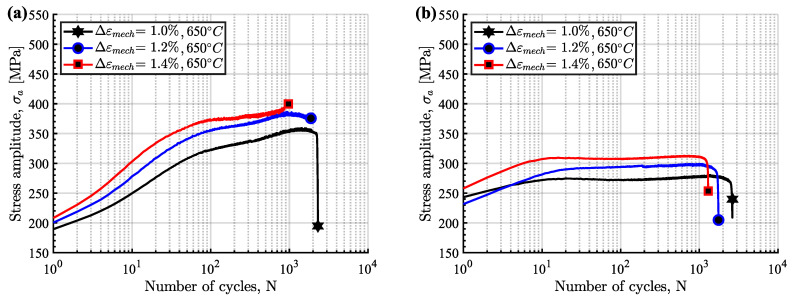
OP-TMF hardening curves of Esshete 1250, (**a**) unaged, (**b**) aged.

**Figure 8:**
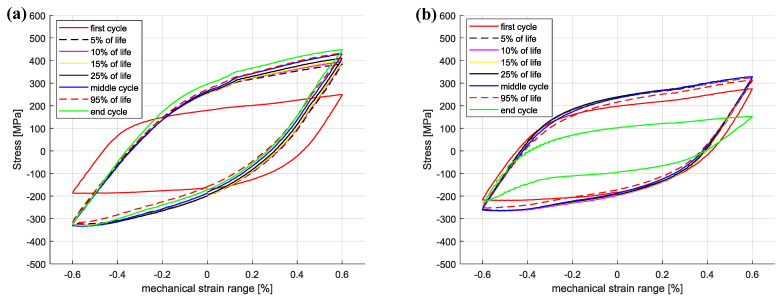
OP-TMF hysteresis curves of Esshete 1250, ΔT = 100–650 ${}^{\circ }$C, $\Delta \varepsilon_{mech}$ = 1.2%, (**a**) unaged, (**b**) aged.

**Figure 9:**
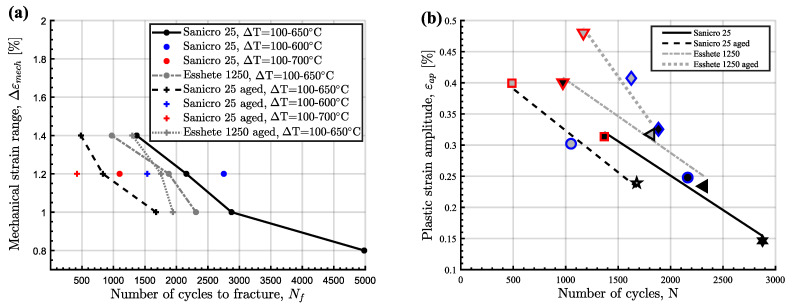
Summation plots of the two investigated materials, (**a**) OP-TMF life of the tested specimens, (**b**) The plastic strain amplitude at OP-TMF midlife in relation to the amount of cycles to fracture, ΔT = 100–650 ${}^{\circ }$C.

As for the unaged Sanicro 25 specimens, the cyclic hardening/softening curves of the Esshete 1250 unaged specimens (Figure 7a) show initial hardening with some saturation for all tests and softening for the tests with the lower $\Delta \varepsilon_{mech}$. For the aged condition (Figure 7b), the amount of hardening decreased and the specimens experienced longer saturation at lower stress levels, with succeeding softening until the failure criteria was reached. As a consequence of this, the aged specimens also showed increased plastic strain range (Figure 8b), which did not decrease after the initial stage as for the unaged specimens (Figure 8a). With greater applied $\Delta \varepsilon_{mech}$, the ageing did not clearly negatively affect the OP-TMF life of Esshete 1250 as it did for Sanicro 25 (Figure 9a). Although, the loss of structural integrity induced higher plastic strain range at lower stress levels (Figure 9b). Another indication of this is the Vickers hardness results in Figure 6, where the tested aged specimens show lower hardness than the equivalent tested unaged specimens. All the undeformed specimens have similar hardness level.

**Figure 10:**
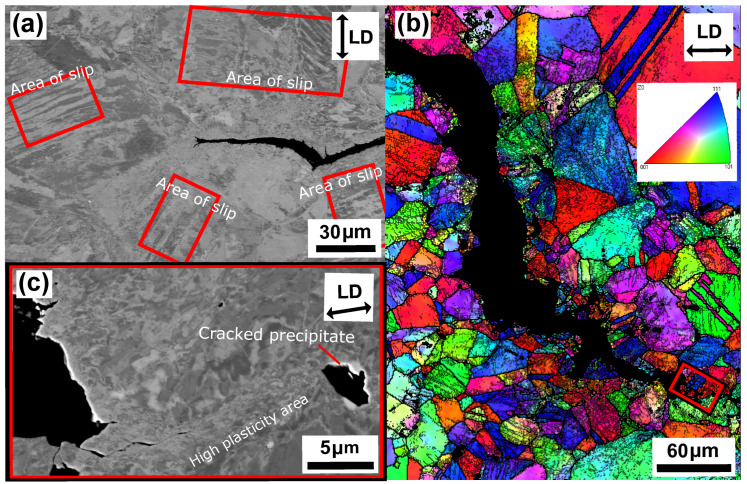
Micrograph of unaged Sanicro 25, ΔT = 100–650 ${}^{\circ }$C, $\Delta \varepsilon_{mech}$ = 1.2%, (**a**) BSE analysis of secondary initiation overview, (**b**) EBSD analysis of the main crack, (**c**) enlarged BSE image of the crack tip in (**b**).

**Figure 11:**
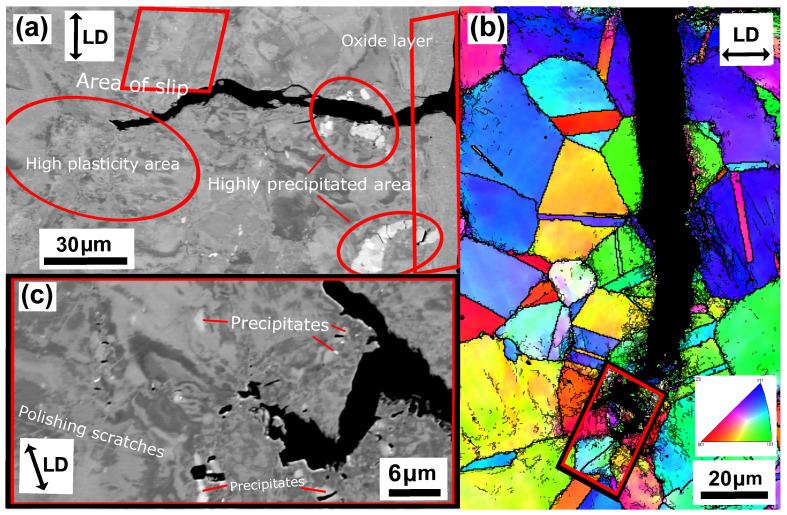
Micrograph of aged (T = 650 ${}^{\circ }$C) Sanicro 25, ΔT = 100–650 ${}^{\circ }$C, $\Delta \varepsilon_{mech}$ = 1.2%, (**a**) BSE image of a secondary initiation overview, (**b**) EBSD analysis of a secondary crack initiation, (**c**) enlarged BSE image of the crack tip in (**b**).

**Figure 12:**
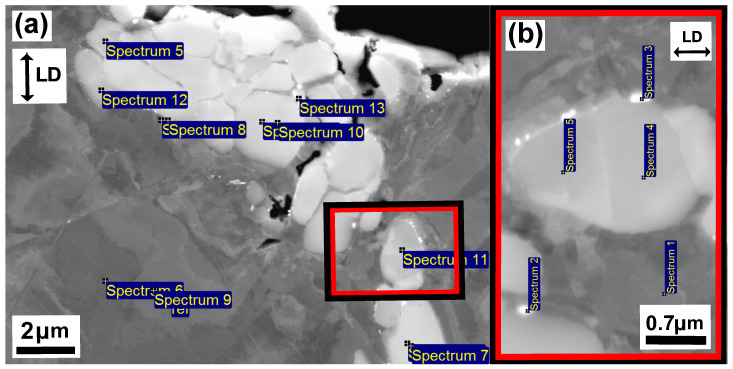
Micrograph of aged Sanicro 25, ΔT = 100–650${}^{\circ }$, $\Delta \varepsilon_{mech}$ = 1.2%, (**a**) BSE image which show the EDS point spectrum positions of the highly precipitated region in the middle of the crack in Figure 11a, (**b**) enlarged BSE image which show the EDS point spectrum positions (zoomed image in (**a**)).

Figure 10 and Figure 11 show the microstructure of unaged and aged Sanicro 25 after OP-TMF testing ($\Delta \varepsilon_{mech}$ = 1.2%). The unaged specimens did not proceed to complete fracture (complete specimen break) and the main crack seemed to have been propagating transgranularly according to Figure 10b. Many of the bulk material grains show signs of plastic straining, where areas of severe plastic deformation can be seen at the vicinity of the cracks (Figure 10a,b) and other grains show what appears to be slip formations (Figure 10a). Plastic straining in the form of slip formations are frequently observed during high temperature loadings for these kind of materials [20,21]. Figure 10c shows a magnified crack tip area of the main crack, which contains high plasticity and precipitates enriched by nitrogen, chromium, and niobium that either cracked during straining or have been detached during the polishing/grinding of the sample. By comparison, the aged Sanicro 25 condition show similar “cracked” precipitates, but in larger sizes and quantities and sometimes precipitates are grouped together occupying relative big areas (Figure 11a and Figure 12), with the bigger precipitates in the middle and with smaller precipitates at its boundaries. With EDS it is hard to identify these grouped precipitates given the resolution of the technique, but these areas show enrichment of niobium, chromium, carbon, nitrogen, copper, and tungsten, as evident by the spectrum quantitative results in Table 2. Similar plastically deformed grains close to the crack initiations and slip formations in the interior of the material can be seen for the aged specimens as for the unaged specimens. However, the aged specimen suffered full fracture (complete specimen break) but the analysis of the crack propagation characteristics was deemed similar to its secondary initiations of Figure 11a,b, which show transgranular crack characteristics.

**Figure 13:**
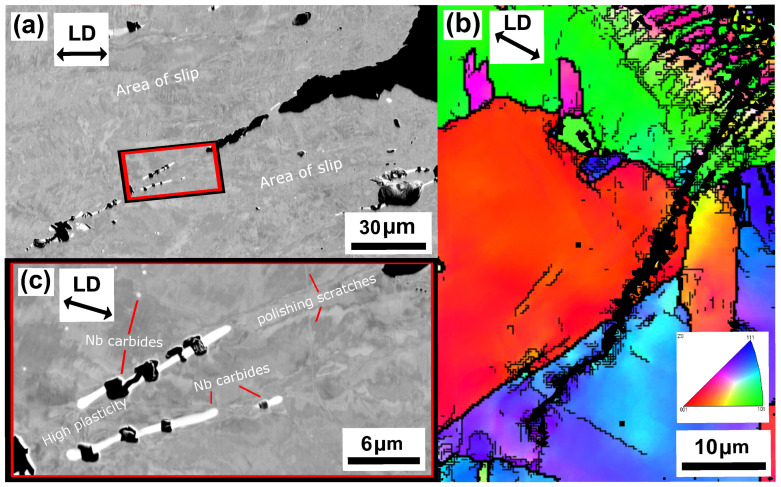
Micrograph of unaged Esshete 1250, ΔT = 100–650 ${}^{\circ }$C, $\Delta \varepsilon_{mech}$ = 1.2%, (**a**) BSE image of crack branching from the main crack, (**b**) EBSD analysis of a secondary crack initiation, (**c**) enlarged BSE image of the crack midsection in (**a**).

**Figure 14:**
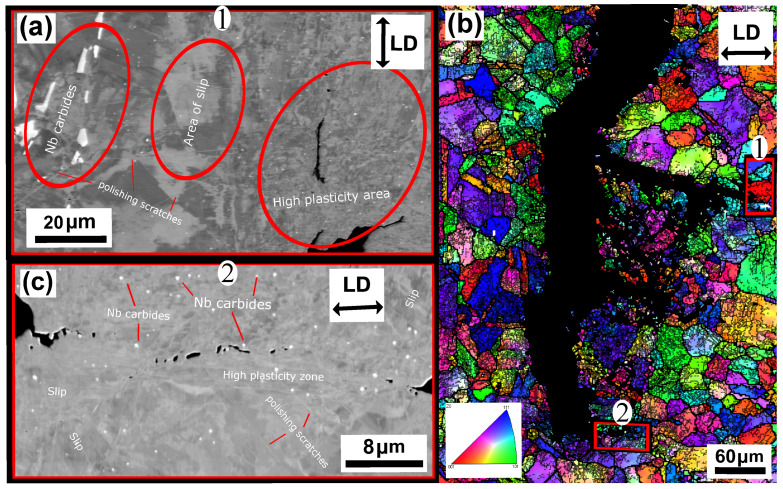
Micrograph of aged (T = 650 ${}^{\circ }$C) Esshete 1250, ΔT = 100–650 ${}^{\circ }$C, $\Delta \varepsilon_{mech}$ = 1.2%, (**a**) BSE image of crack branching from the main crack (zoomed image 1 in (**b**)), (**b**) EBSD analysis of the main crack initiation, (**c**) enlarged BSE image of the crack tip (zoomed image 2 in (**b**)).

**Figure 15:**
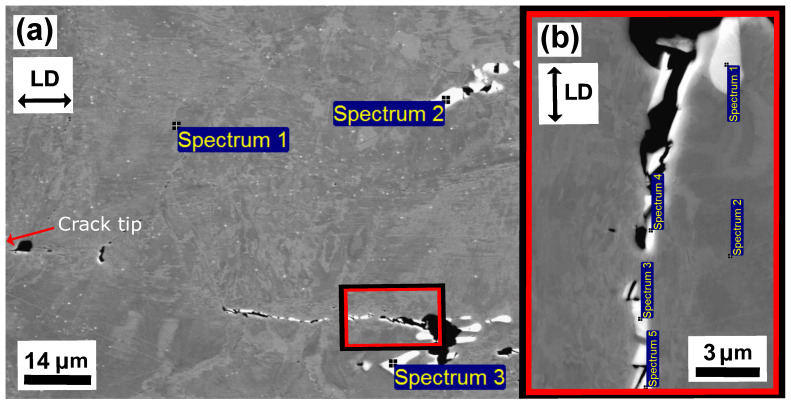
Micrograph of aged Esshete 1250, ΔT = 100–650${}^{\circ }$, $\Delta \varepsilon_{mech}$ = 1.2%, (**a**) BSE image which show the EDS point spectrum positions of the region in front of the crack tip (the area in front of Figure 14c,“(2)”), (**b**) enlarged BSE image which show the EDS point spectrum positions (zoomed image in (**a**)).

The unaged Esshete 1250 $\Delta \varepsilon_{mech}$ = 1.2% specimen, also suffered complete fracture (complete specimen break) and, similar to the Sanicro 25 specimens, the probable propagation type is also transgranular. This is found when investigating secondary initiations, such as in Figure 13b. Figure 13a shows a crack branch connected to the fracture surface of the main crack. Both sides of the crack branch give evidence of plastic straining, most likely in the form of slip areas, which supports crack growth. The close-up image of the crack branch midsection, shown in Figure 13c, indicates that the crack propagation is also influenced by cracking of precipitates. The corresponding aged specimens exhibit areas of the same precipitates that were identified as niobium carbides (NbCs) given the quantitative data in Table 3. In addition, similar evidence of severe plastic straining close to the crack tips can be seen in Figure 14a–c and Figure 15a,b. However, in contrast to the unaged specimens, the aged specimens did not suffer full fracture (complete specimen breakage). Instead, there were one main crack with crack branching and comparably fewer secondary initiations. These are presented in Figure 14b.

## 4. Discussion

The influence of ageing on the mechanical response during OP-TMF of Sanicro 25 is presented in Figure 3, Figure 4, Figure 5 and Figure 6 and Figure 9. The appreciable trend of these graphs is that increased plastic deformation yields shorter cyclic life, even though the stress levels of corresponding test conditions are quite similar. The hardening ability is not lost for the aged specimens, as can be seen in Figure 3a, but comparing Figure 4a with Figure 4b and studying Figure 9b it is clear that the aged specimens suffers additional plastic deformation compared to the unaged specimens. This also applies for different ageing and testing temperatures, as can be seen in Figure 5. The hardening ability of Sanicro 25 has been studied before [18,22,23] and these studies conclude that hardening and high temperature strength are mainly due to the precipitation of Cu-rich nanoparticles and Nb-rich precipitates during cycling at elevated temperature. These impede the dislocation motion during high temperature cyclic straining, where the Cu-rich nanoparticles are homogeneously spread and coherent with the austenitic matrix. However, the key contribution to the obstruction of dislocation movement is from the NbC, NbN, and Nb(C,N) precipitates, which acts as incoherent dispersoids. Ageing and prolonged high-temperature cycling affects the size, distribution, and structure of the precipitates. For Sanicro 25, previous studies have found that Z-phase (CrNbN), $Cr_{2}N$, NbC, and $M_{23}C_{6}$ precipitates are present in the bulk material and at grain boundaries in the as-received state and when aged, needle shaped laves phase, ${\left(Cr,Fe\right)}_{2}W$ (more common at $T_{max}$ ≥ 700 ${}^{\circ }$C), forms and the original precipitates or/and phases increase and coarsen [18,24,25]. This corresponds well with the findings of Figure 10, Figure 11 and Figure 12 for the bigger precipitates. The increased plastic strain range and lower cyclic life of the aged specimens correlates to the reduced ability for impeding of dislocation movement and the reduced solid solution strengthening by the loss of solute atoms as a consequence of the precipitation process. The microstructural evolution during ageing and cycling are accelerated compared to the unaged condition and promotes the decrease, restructuring, and coarsening of the strengthening precipitates, as well as formation of new detrimental or not as effective precipitates or/and phases. For both conditions, the crack initiations are facilitated by cracking of the surface oxide layer [14,16] and then transgranular propagation through the bulk material with accompanied plastic deformation (Figure 10a,b and Figure 11a,b). Whether or not precipitate cracking accelerates the crack propagation is hard to determine because of the lack of real-time (in-situ) or interrupted testing. The “cracked” precipitates found in Figure 10c, Figure 11c and Figure 12 are located in areas of significant plastic deformation and cracking of these could provide a privileged crack path and accelerate failure. However, they could also be considered holes surrounded by element enrichment that have been ground out during the grinding and polishing treatment. However, damage could potentially nucleate at the interface between the matrix and precipitate during high straining, which could coalesce with the crack initiation and promote growth [26,27]. Other studies have reported oxidation and creep to assist cracking during TMF testing of austenitic stainless steel, as mentioned in the introduction [12,14,16,17,18]. Creep damage is more likely promoted in IP-TMF during tensile stresses at high temperature in the form of dislocation climb (high stresses) or cause diffusion at the grain boundaries (lower stresses) that eventually cause formation of micro cracks. Oxidation mechanisms can affect both IP- and OP-TMF. For IP-TMF, an oxide film forms during tension at high temperature and then breaks because of buckling upon cooling in compression, new material is then exposed that can be oxidised and later break so that a repetitive process will induce a crack. For OP-TMF the oxide layer is instead formed during compression and breaks when the material is mechanical strained during cooling. During the investigations of present study, no concrete evidence of oxidation assisted cracking were observed other than crack initiations at the surface. These were formed as a consequence of surface oxide layer deformation due to the thermomechanical cycling, which frequently is the initial damage process during OP-TMF and have been observed in other investigations [11,14,16]. The propagation in the bulk was however considered fatigue dominated.

According to Figure 9a the OP-TMF life of the reference material, Esshete 1250, does not show a clear trend when the material is aged before OP-TMF testing. However, when studying Figure 6, Figure 7 and Figure 8 and Figure 9a, it can be concluded that the ageing greatly affects the plastic behaviour of the material. The hardening is greatly reduced (Figure 7) and the plastic strain range is generally higher for all the aged specimens. Given this and that the used OP-TMF method was strain controlled, it was possible for the aged specimens to reach the designated $\varepsilon_{mech}$, but for lower stress levels. This did not trigger the load drop failure criteria because the internal resistance to straining was at a steady state of yielding. Considering the different microstructural evolution of the two conditions in Figure 13, Figure 14 and Figure 15, the crack propagation seems to be associated with cracking of the niobium carbides (NbCs) and the surrounding area exhibit noticeable high plastic deformation. For the aged specimens, the NbCs were coarser and grouped together in certain areas and this should be the reason for the lowered structural integrity [28]. Given that the unaged specimens had the higher structural integrity and, therefore, suffered higher stresses when cycled to the predetermined $\varepsilon_{mech}$ level, locally a high enough stress level could be reached for cracking of the NbCs, which would assist the crack propagation. This is considered to be an explanation to the more brittle failure characteristics (Figure 7a) involving complete fracture (total breakage of the specimen) rather than triggering of the load drop failure criteria for the unaged Esshete 1250.

## 5. Conclusions

The OP-TMF behaviour of two austenitic stainless steels, Sanicro 25 and Esshete 1250 have been investigated. The Sanicro 25 results show that the aged specimens suffered increased plastic straining and shorter OP-TMF life compared to the unaged specimens. The difference in OP-TMF life of the two test conditions was attributed to an accelerated microstructural evolution that provided decreased effectiveness for impeding dislocation motion. There was not a similar clear trend for the reference material Esshete 1250 considering the unaged and aged specimen OP-TMF lives. However, the structural stability and the resistance for cyclic deformation was greatly reduced due to coarsening and cracking of the strengthening niobium carbides.

## Figures and Tables

**Table 1:** Chemical composition (in wt.%) of the austenitic alloys (provided by Sandvik Materials Technology AB).

Material	C	Cr	Ni	W	Co	Cu	Mn	Nb	N	Si	V	Mo	Fe
Sanicro 25	0.1	22.5	25.0	3.6	1.5	3.0	0.5	0.5	0.23	0.2	-	-	Bal.
Esshete 1250	0.1	15	9.5	-	-	-	6.3	1.0	-	0.5	0.3	1.0	Bal.

**Table 2:** The quantitative EDS spectrum results (in wt.%) of the aged Sanicro 25 microstructure in Figure 12.

Spectrums	C	Cr	N	Ni	Cu	Nb	W
Figure 12a							
Spectrum 5	6.7	25.7	4.2	5.2	0.7	44.5	3.6
Spectrum 6/9 (ref)	3.3	22.2	0.5	24.0	2.8	0.4	3.6
Spectrum 7	5.9	27.0	3.7	0.6	0.2	57.5	4.3
Spectrum 8	6.7	24.0	0.9	14.1	7.5	16.9	4.7
Spectrum 10	6.9	26.9	4.4	1.1	0.2	56.3	3.8
Spectrum 11	6.0	27.9	5.6	2.2	0.3	52.5	3.5
Spectrum 12	5.3	23.3	1.5	12.3	1.4	29.0	4.0
Spectrum 13	7.2	25.3	2.4	4.3	0.6	47.8	4.6
Figure 12b (enlarged image)							
Spectrum 1 (ref)	4.7	20.6	0.4	23.3	3.5	0.7	3.2
Spectrum 2	5.9	20.1	2.5	14.3	1.3	22.9	7.9
Spectrum 3	7.7	24.1	2.1	10.0	1.3	31.6	5.0
Spectrum 4	9.8	25.7	5.2	2.5	0.3	50.0	4.0
Spectrum 5	10	27.9	4.9	2.9	0.4	46.9	2.9

**Table 3:** The quantitative EDS spectrum results (in wt.%) of the aged Esshete 1250 microstructure in Figure 15.

Spectrums	C	Cr	Mn	Fe	Ni	Nb	Mo
Figure 15a							
Spectrum 1 (ref)	2.9	15.4	7.2	61.0	9.8	0.9	1.4
Spectrum 2	16.4	4.1	1.6	12.9	1.6	62.7	0.1
Spectrum 3	6.7	11.1	4.6	43.6	6.1	26.3	0.9
Figure 15b (enlarged image)							
Spectrum 1	18.3	1.4	0.5	3.3	0.41	75.1	0.3
Spectrum 2 (ref)	2.8	15.3	6.7	63.5	9.2	0.5	1
Spectrum 3	5.56	17.0	5.5	47.9	6.9	14.7	1.7
Spectrum 4	4.21	13.2	6.1	58.8	8.3	7.5	0.9
Spectrum 4	4.5	13.1	5.9	55.8	8.0	10.8	0.9
